# Meta-Analysis of the Structural Equation Models' Parameters for the Estimation of Brain Connectivity with *f*MRI

**DOI:** 10.3389/fnbeh.2018.00019

**Published:** 2018-02-15

**Authors:** Joan Guàrdia-Olmos, Maribel Peró-Cebollero, Esteve Gudayol-Ferré

**Affiliations:** ^1^Department of Social Psychology and Quantitative Psychology, School of Psychology, Institute of Neuroscience, Institute of Complexity, University of Barcelona, Barcelona, Spain; ^2^School of Psychology, Universidad Michoacana de San Nicolás de Hidalgo de Morelia, Morelia, Mexico

**Keywords:** fMRI, structural equation models, functional connectivity, effective connectivity, cognitive neuroscience

## Abstract

Structural Equation Models (SEM) is among of the most extensively applied statistical techniques in the study of human behavior in the fields of Neuroscience and Cognitive Neuroscience. This paper reviews the application of SEM to estimate functional and effective connectivity models in work published since 2001. The articles analyzed were compiled from Journal Citation Reports, PsycInfo, Pubmed, and Scopus, after searching with the following keywords: fMRI, SEMs, and Connectivity.

**Results:** A 100 papers were found, of which 25 were rejected due to a lack of sufficient data on basic aspects of the construction of SEM. The other 75 were included and contained a total of 160 models to analyze, since most papers included more than one model. The analysis of the explained variance (R^2^) of each model yields an effect of the type of design used, the type of population studied, the type of study, the existence of recursive effects in the model, and the number of paths defined in the model. Along with these comments, a series of recommendations are included for the use of SEM to estimate of functional and effective connectivity models.

## Introduction

Structural Equation Models (SEM) have been among the most extensively applied statistical techniques in the scientific literature in the last 30 years. Since their first description (Jöreskog and Sörbom, 1979, 1984), they have been widely used in both the social sciences and the health sciences, and also in the study of human behavior based on the precepts of the wide domain of Neuroscience and Cognitive Neuroscience and the latest contributions of Computational Quantitative Neuroscience. It has been argued that the specification of a given structural model with a theoretical basis allowed the estimated parameters to assume the effect of the impact of exogenous variables on endogenous variables, which has more recently been referred to as neuroscience hypothesis generation (Lange et al., 2013). We do not intend to offer a comprehensive description of the properties and characteristics of SEMs, but the reader has an excellent base at their disposal on Bollen and Scott-Long (1993), Brown (2006), Raykov and Marcoulides (2006), Byrne (2010), Everitt and Hothorn (2011), or Kline (2011). Generally speaking, it is a multivariant technique intending to estimate structural relations between latent variables generated from observable variables. Such structural relations allow us to identify, through a system of simultaneous linear equations, whether one possible theoretical model can be confirmed through the distributions observed in the variables involved in that model. As for the papers on functional or structural connectivity with an fMRI signal, this type of model allows us to identify the relations and their statistical estimation among brain areas, both in a resting state or upon a cognitive task.

All this has affected the approximations established through SEM for the estimation of functional and effective brain connectivity from a brain signal registered by increasing the *Bold* (Blood Oxygen Level Dependent) signal under experimental paradigms with cognitive content in *f* MRI (functional Magnetic Resonance Image) situations (see the description by Price, 2012). The use of SEM in this specific domain has been so important that we feel the moment has come to assess the use made of the technique for the study of brain connectivity. The earliest relevant contributions to this subject can be found, mainly, in the work by McIntosh, and Gonzalez-Lima (1991); McIntosh and Gonzalez-Lima (1992), while certain guidelines appear in McIntosh and Gonzalez-Lima (1994) for the use of SEM for the estimation of brain connectivity. The latter paper defines some interesting concepts. The basic conception lies in the fact that their computation capacity allows us to identify cognitive processes as a complex series of hierarchically organized computational models. Evidently, this conception fits perfectly with the statistical formulations of SEM. It is assumed, moreover, that the processes analyzed are usually conceived as separable and that the final cognitive process is defined by the adding together of the partial processes. The above comments clearly define the concept of functional connectivity, given that the estimation of SEM is generated, in this case, without considering the biological structure of the nervous system. Therefore, we speak of functional connectivity to refer to statistical models formulating stochastic structural relationships between specific brain regions of interest (ROI's) that show statistically significant activity when facing certain cognitive content tasks. McIntosh (1999) describes the following phases for estimating functional or effective connectivity through SEM: (a) selecting regions or nodes of the network driven by a combination of univariate analysis of changes in signal (*f* MRI) intensity, multivariate analyses, and theoretical guidance; (b) obtaining the anatomical model, that is, clearly identifying the fact that the regions selected in the previous stage are coherent with the functionality attributed to the ROI selected; (c) calculating the interregional covariance or correlations matrix from the *f* MRI data. These matrices can be computed for an individual subject across tasks or across trials of the same task; and finally (d) calculating the path coefficients and comparison of functional models according to the characteristics of the statistical estimation technique and the properties of the distributions observed. The second of these phases deserves special attention since today there is a certain balance between several mechanisms for selecting ROIs to analyze. Currently, all the possible effects that can be established between the ROIs detected in previous univariate or multivariate analyses are often specified. Occasionally, these effects have no neurofunctional support and are justified solely by statistical effects. Accordingly, the second phase becomes a mixture of known neurofunctional effects and a certain exploration of effects based on previous statistical significances. In fact, some authors have proposed that ROI selection based only on statistical criteria can lead to certain circular fallacies and to effect overestimation (Farràs-Permanyer et al., 2015). A well-known example is the “double dripping” effect, described by Kriegeskorte et al. (2009) which occurs when orthogonal contrasts are not properly established in the first phase of the statistical analysis that identified statistically significant ROIs. In addition, questions associated with the ability of correlations or covariance estimates to reveal connections between brain regions remain unresolved in the sense that, as is well-known, estimates of correlations are not usually taken into account in this type of work (Marrelec et al., 2009; Vul et al., 2009).

This initial guide to fitting SEM to the estimation of brain connectivity has been complemented by many contributions, especially statistical ones, which have led this topic toward slightly more complex schemes of action (Penny et al., 2004; De Marco et al., 2009; Kim and Horwitz, 2009; Penke and Deary, 2010; Rowe, 2010, or Schlösser et al., 2006). Likewise, some alternatives to the general model of SEM have been developed, as well as some occasional contributions which have generated interesting statistical approximations to the study of connectivity. Some of them are within the logic of SEM, like Unified Structural Equation Models (uSEM) (Chen et al., 2011; Gates et al., 2011; Gates and Molenaar, 2012; Moreira et al., 2016), which involves estimating SEM parameters by means of a two-stage technique (that propose a first step consisting in a reparameterization of the originals structural parameters to avoid the collinearity effects and a second step to estimate through Ordinary Least Square the parameters free of this perturbation) and which proposes the use of auto-regressive vectors; several choices of structure and estimation like the Extended Unified Structural Equation Models (euSEM) (Gates et al., 2010; Taylor et al., 2010), which adds to uSEM the possibility to specify direct effects representing the effect of manipulating stimuli in event-related designs, along with the more recent scheme by Inman et al. (2012) or elements connected with the circular complex analysis (Kriegeskorte et al., 2010; Sato et al., 2014) which shows the most consolidated phases in the generation of SEMs for the estimation of functional connectivity today (Carp, 2012).

### Structural equation models in fMRI complex analysis

The SEMs applied in this field are based on the so-called type-III models and are identified by the general expression

$$\begin{array}{l} y_{t}=\beta y_{t}+\zeta_{t}, \end{array}$$

where *y*_*t*_ are the values of the ROIs selected, β the parameter estimations, and ζ_*t*_ the structural errors associated with each endogenous variable *y*_*t*_. Each ROI is the result of generating a score (*y*_*t*_) for each brain volume. This score is calculated through the regression equation established with the values of the voxels which define it by means of a unidimensional principal components analysis. In these terms, we should bear two aspects in mind. Whatever the parameter estimation technique (usually Maximum Likelihood ML), the statistical problem involves estimating the parameters of the β matrix that encompass the effects between ROIs, and the ψ matrix's parameters that encompass the matrix of the variances/covariances between the ζ_*t*_ structural errors, so that ψ = *E(*ζζ′*)*. The specific form that β adopts derives from the aforementioned effects between ROIs, and the form that ψ adopts summarizes the assumptions specified according to the distributions of the structural errors. Generally, the classical assumptions of SEM would involve the initial assumption that *E(*ζζ′*)* = *E*($y_{t}\zeta^{\prime }$*)* = 0 and, consequently, the errors should be uncorrelated between themselves in relation to the endogenous variables, except for the possibility that the β matrix considers non-recursive effects (recursive models imply that the connection between two ROIs can only go in one direction, while non-recursive models facilitate reciprocal connections between ROIs). Evidently, this enables the error distribution to be independent of the β estimations. This model also involves the assumption that the variables, i.e., the values for each ROI, are observed continuous variables of a multinormal distribution. The truth is that each value of *y*_*t*_ representing the *f* MRI of a ROI is estimated through Principal Component Analyses (less often a peak voxel and averaging within sphere approach is also used) according to the selection of a specific number of voxels convoluting under a geometric form (generally a sphere) defined around a voxel of maximum statistical significance, univariate or multivariate, under the statistical assumptions of the massive general linear model. Therefore, every *y*_*t*_ extracted could be considered a latent variable (η_*t*_) defined based on the actual observed values in each voxel and estimated according to the type of design used (Block Design or Event-Related Design) or in some cases, from a series of *f* MRI data registered in a resting state paradigm.

This issue is rather controversial, and there has been discussion about whether functional or effective connectivity is established through Path Analysis (PA) for observable variables and which does not allow non-recursive effects (reciprocal influence between ROIs), or whether it is strict SEM with latent variables (though not specified as such) and which allows non-recursive effects. To clarify this matter, we should note that the techniques based on PA only admit observable variables, whereas SEM accept both observable and latent variables. Therefore, the final scores of each ROI can be considered as latent. Thus, complex SEM models are essential to the study of connectivity, given that it would be sensible to include the existence of reciprocal effects between ROIs and to estimate what Berry (1984) called complete reciprocity models, that is, specifying all the possible reciprocal effects between variables, or ROIs in this case. This idea is indirectly repeated in the scheme by Inman et al. (2012).

**Figure 1:**
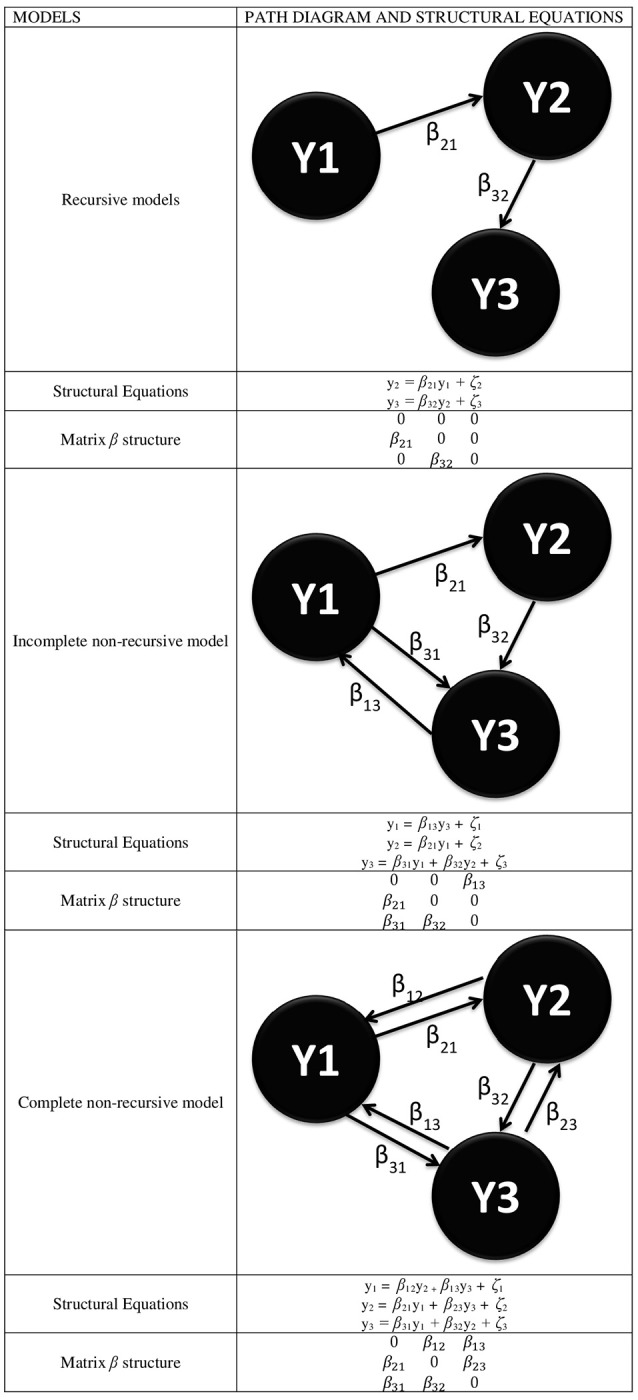
Different types of SEMs for the representation of functional connectivity where every YI represents a ROI and with the specification of the structural equations linked to every model and the specific form of the β matrix.

From the point of view of SEM, there are basically two possible structures that can be submitted to an analysis to represent connectivity models: non-recursive and recursive structures, in the latter case either complete or incomplete. Figure 1 shows a simple diagram (with only three ROIs) of both possibilities, with an indication of the structural equations associated with each model.

From this figure, it is inferred that the β matrix can adopt several forms and that both the decomposition of effects and the resulting value of the estimations depend on it. Likewise, the above comment on the *E(*ζζ′*)* = 0 assumption yields a ψ diagonal matrix if assumed, or a ψ symmetrical matrix if unassumed, given that *E(*ζ_*i*_ζ_*j*_*)* = *E(*ζ_*j*_ζ_*i*_*)*. Also, the β matrix can adopt a triangular form in the recursive models or any form in the non-recursive models, as long as the model identification problem is solved by justifying that *E(*ζζ′*)* = 0.

These and other matters are not usually dealt with in papers on connectivity and have been solved in different ways. In recent years, many papers have been published in this field applying different statistical approximations to the topic. Sometimes they are not even mentioned, and sometimes the information provided is only partial. The first consequence of this is an extraordinary variety of approaches and statistical treatments, a diversity which occasionally compromises the comparability of results between papers and even a reasonable deduction regarding the functional conception of connectivity. In fact, SEMs present a series of restrictions that are linked to their own statistical structure and that of the associated assumptions which, in most of the fields in which they are applied, are ignored or, at least, no evidence is provided to the contrary. For example, in the field of Social Science and Health Science, in general, there are no data that prove that the model's assumption was complied with: for instance, the distributions of the observed variables, or the order or range conditions, especially in non-recursive models where both conditions could be seriously compromised. This is a situation that can be resolved by the appearance of computer software (Amos, MPlus, or sem library of R), since it incorporates several guarantees. However, little is said about it in model presentations, just as little, or nothing at all, is said of the statistical assumptions. For instance, as shown above, assuming or not assuming that *E(*ζζ′*)* = *0* leads us to a very different consideration of the β matrix and of the possible effects to assess. Some reviews have been published on this topic, so we know a certain amount about the limitations of the concept of functional or effective connectivity and its results in relation to its neurobiological parallel. If we look at some papers (James et al., 2009; McCormick et al., 2010; Bianchi et al., 2012; Deshpande and Hu, 2012; Murray et al., 2012; Sawyer et al., 2012; Voineskos et al., 2012; Yang et al., 2012; Bringmann et al., 2013) we find that all of them account for the limits of the concept of connectivity, of its possibilities in relation to effective connectivity, and the derivatives obtained from using it from an applied perspective; but little, if anything, is said about good practices in the use of SEMs and their adaptations for the statistical estimation of brain connectivity.

In view of all of the above, the present paper aims to review the application of SEM for the estimation of connectivity models in work published since 2001. By doing so, we mean to establish the effect of several variables pertaining to studies on connectivity with *f* MRI signal on the estimation of the *R*^2^ (Coefficient of Determination representing the proportion of explained variance) each model presents. This way we intend to break through in the systematization of some of the statistical properties in their application and some of the characteristics of SEMs in the field of Computational Neuroscience when generating estimations that lead us to the consideration of a globally-analyzed functioning brain, and when generating models from complexity. At the moment we have no evidence of the possible effect on *R*^2^ of variables like the type of design used, the number of ROIs defined, the parameter estimation technique used, or the type of sample analyzed, among others. We also aim to offer some recommendations to future users of SEM in this field in order to generate, in the near future, a good mechanism for comparing the results for functional or effective connectivity obtained in different studies.

## Materials and methods

### Search for studies

**Figure 2:**
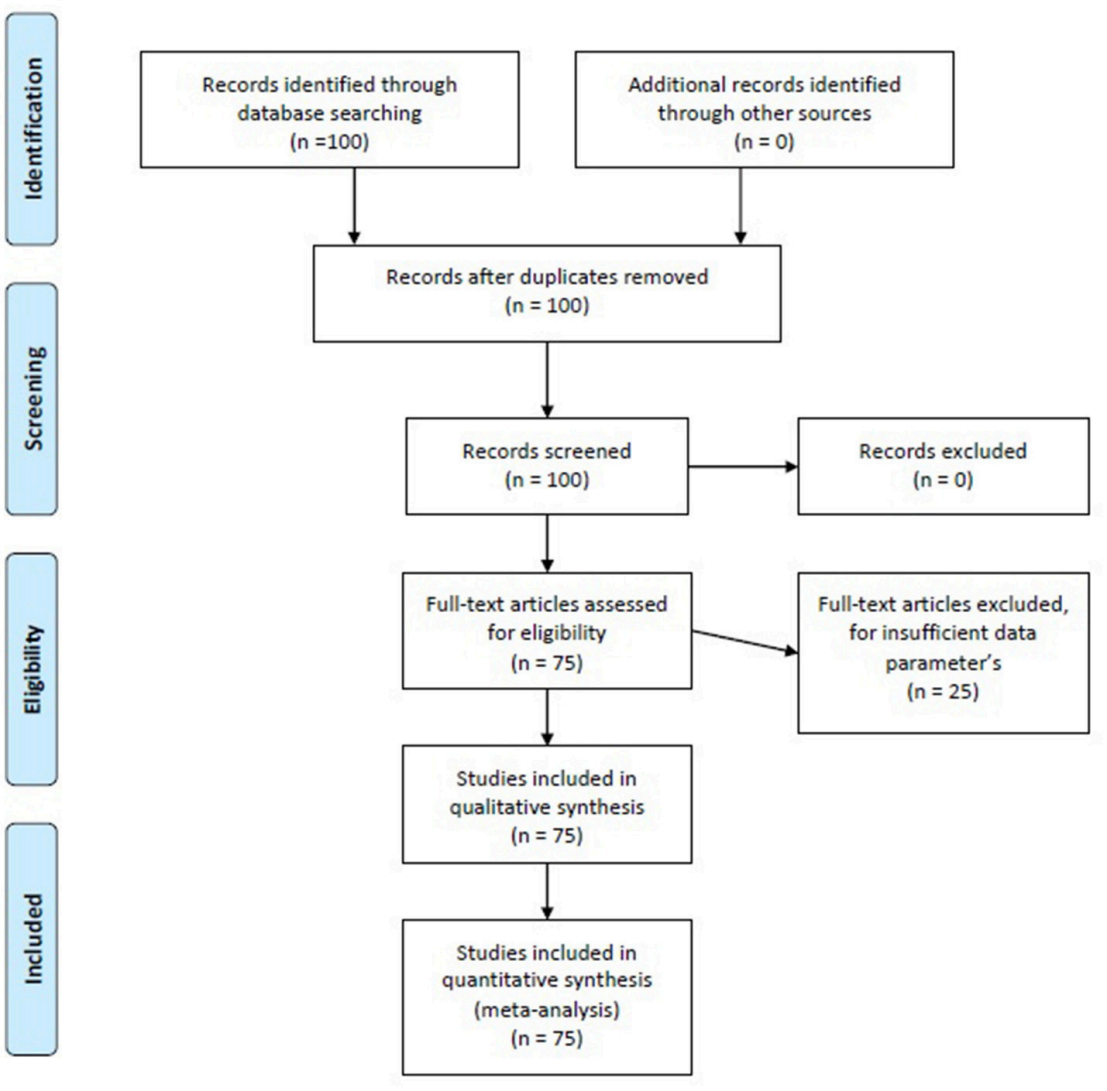
Flow Chart of the bibliography search.

To be included in the present meta-analysis, the articles had to comply with the following criteria: (a) they had to be original *f* MRI papers approaching a topic of brain connectivity using a data analysis technique directly related to SEM (so we selected all the papers whose data had been analyzed through SEM, PA, uSEM, and euSEM; (b) each model had to be estimated in a different group or sample, so that even if one paper presented multiple models, each one would be estimated in different samples; (c) they had to have been published between 2001 and 2016; (d) they had to be indexed in Journal Citation Reports, PsycInfo, Pubmed, or Scopus; and (e) they had to explicitly offer β matrix standardized parameter values for each model analyzed and the initial matrix *R* (Correlation Matrix) or *S (Covariance-Variance Matrix)* (some authors kindly provided us with this information, since it did not appear in the original published papers). The search for papers was conducted by means of a Boolean algorithm using the following keywords: “*f* MRI,” “Structural Equation Models,” and “Connectivity.” Studies listed in more than one of the aforementioned sources were not duplicated. With the general selection of the aforementioned keywords, we found 100 papers, 25 of which were rejected due to a lack of sufficient data on basic matters regarding the construction of SEM such as, for example, presenting the values of the specific parameters or not offering multiple equation models, using uni-equation models similar to multiple regression models. Eventually, after two independent reviews of this process, 75 papers were included (available in Data Sheet 1 of the Supplementary Material) containing a total of 160 models to analyze, given that most of them included more than one model (*M* = 1.47; *SD* = 1.06), ranging between one and eight models per paper]. The process of including and excluding papers is shown in Figure 2.

### Coding of the variables

To each of the papers selected, we applied a template with which we obtained the values of the different variables assessed in this study by two independent researchers. As has just been mentioned, it should be noted that the majority of papers included more than one structural model, so the data were generated one model at a time; therefore, the number of models is much higher than the number of selected articles. For each model, we registered the variables listed in Tables 1, 2.

**Table 1:** List of categorical variables according to their characteristics and codifications.

**Variables**	**Variable's consideration**
Year of publication (2001–2013)	Context
Journal	Description
Technique used (SEM, PA, uSEM, euSEM)	Methodological
SEM: Structural Equation Model	
PA: Path Analysis	
uSEM: Unified SEM	
euSEM: Extended Unified SEM	
Type of design (Box Car one group, Box car two groups, Box car more than two groups, Simple event related, Complex event related, Conjunction design, and Resting state paradigm)	Methodological
Note: Except for the resting state situation, the rest of designs must include some kind of periodical cognitive stimulus. Resting state designs do not include any kind of stimulus	
Strategy of Comparisons (Between Subjects, Between Groups, Between Tasks, and Factorial Task, and groups)	Methodological
Type of population studied (Healthy/Normal, Clinical, Both, and Simulation study)	Methodological
Kind of study (Data driven, Hypothesis driven, and Both)	Methodological
Recursive effects (Yes or No)	Methodological
Estimation technique (ML, WLS, Bootstrap, and Others, No information)	Methodological
Multinormality analysis (Yes or No information)	Methodological
Matrix analyzed (Correlation, Covariance, or No information)	Methodological
Conditions studied (Well-conditioned or No information)	Methodological
*p*-value associated to Chi Square (Inferior to 0.10 or Superior or equal to 0.10)	Methodological
Other fit indexes reported as Comparative Fit Index, Bentler Bonnet Fit Index, Akaike Criteria, etc. (Yes or No)	Methodological

**Table 2:** List of quantitative variables according to their characteristics and codifications.

**Variables**	**Variable's consideration**
Total sample size	Methodological
Clinical sample size	Methodological
Healthy sample size	Methodological
Number of brain areas analyzed	Substantive
Number of defined paths	Methodological
Chi square value	Methodological
*p*-value of chi square	Methodological
Coefficient of Determination (*R^2^*)	Outcome

### Calculating the *R^2^* as effect size

As mentioned, in each of the structural models selected, we identified the *R*^2^-value. In some cases the papers did not report this value explicitly but included the values of the initial *R* or *S* matrix (*R* is the input correlation matrix and *S* is the variance-covariance matrix, both as possible inputs data) and the structure and values of the β matrix; in these cases we estimated *R*^2^ from the available values. In other cases, the contact authors sent us the information mentioned. Following the classical general scheme, and using the classical LISREL notation:

$$\begin{array}{l} Y=\beta Y+\zeta , \end{array}$$

where *Y* is the matrix of values of specific ROIs, β is the matrix of the effects between ROIs, and ζ is the vector of errors associated with each ROI. It should be noted that the estimations of the β_*ij*_ free parameters respond to the so-called path-rule, so that each of the coefficients of correlation between ROIs is decomposed according to the effects established between them in the Structural Model specified. Therefore, the fitting system of a structural model like the one presented here responds to a component of the Σ partitioned matrix that can be represented as follows:

$$\begin{array}{l} \Sigma =E\left(YY^{\prime }\right)=E\left[\left(\beta Y+\zeta \right){\left(\beta Y+\zeta \right)}^{\prime }\right], \end{array}$$

one of the basic expressions in the fit of SEMs, so each *r* original correlation is compared to the r^*^ reproduced value derived from Σ. So each residual is estimated by means of (*r* − r^*^), which are evaluated through a χ^2^-test of fit (depending on the parameter estimation technique used). Accordingly, the residuals can be defined by means of (*R-*Σ) and therefore

$$\begin{array}{l} \text{Residuals}=\left(R-\Sigma \right)=R-\left\{E\left[\left(\beta Y+\zeta \right){\left(\beta Y+\zeta \right)}^{\prime }\right]\right\}. \end{array}$$

An essential aspect to bear in mind in models of this type is the possibility of all the structural errors being either correlated or uncorrelated. The SEM general scheme requires that if *E(*ζζ′*)* ≠ *0*, then the specification possibilities of the β matrix become restricted; whereas if we assume that *E(*ζζ′*)* = *0* and, therefore, the structural errors are uncorrelated, β can adopt several forms and assume non-recursive effects—which, in the case of functional connectivity, is essential. On the other hand, whatever the structure of the β matrix, it does not consider the specification of free parameters in the principal diagonal, so it is assumed that the β_*ii*_-values are fixed. This is one of the aspects that presents statistical differences with regard to the effective connectivity models based on Dynamic Causal Models. So, if we assume, as is usual in these models, that *E(*ζζ′*)* = *0* and, by extension, that *E(*βζ′*)* = *0*, the above expression can be rearranged so that

$$\begin{array}{l} Residuals=R-E\left(\beta \beta^{\prime }\right), \end{array}$$

thus obtaining the expression of the reproduced matrix Σ as follows:

$$\begin{array}{l} \Sigma =E\left(\beta \beta^{\prime }\right). \end{array}$$

Therefore, the estimation of *R*^2^ for each model was obtained by calculating the proportion of variance explained by the following simple calculation, standardizing all the values of the initial var-covar matrix (*S*)

$$\begin{array}{l} R^{2}=tr\left(\Sigma \right)/tr\left(R\right). \end{array}$$

We consider this to be a robust indicator of the effect between ROIs and therefore available for use as an effect size estimator (Vesterinen et al., 2014). Finally, in the models that do not include the *p*-value associated with the χ^2^ contrast of fit, this value was reproduced based on the distribution model and the degrees of freedom reported for each model analyzed.

### Statistical analysis

Finally, these values were analyzed following the scheme used by Redondo et al. (2002) adapted to our main objective, with the exception that, in our case, we eventually opted for a random-effect model, given the high variability of the observed distributions of the parameters considered as effect sizes. All the analyses were conducted with the IBM-SPSS software, version 23.0, and with some of the R software routines—more specifically, the Meta library (Schwarzer, 2013) and Mplus version 7.4.

## Results

Several different phases of result analysis were carried out due to the amount of variables and data evaluated. First we described all the variables evaluated and, based on these results we selected the ones that provided relevant data. Then, we analyzed inferentially the effect of each variable on the values of the outcome variables defined.

### Description of the results of each model analyzed

Tables 3, 4 show the values of observed distributions for each variable according to the lists in Tables 1, 2 above.

**Table 3:** Statistical descriptives of qualitative variables.

**Variables**	**Number of models**	**Percentage**
**YEAR OF PUBLICATION**		
2001	2	1.25
2002	3	1.88
2003	4	2.50
2004	12	7.50
2005	5	3.13
2006	12	7.50
2007	14	8.75
2008	5	3.13
2009	22	13.75
2010	20	12.50
2011	21	13.13
2012	14	8.75
2013	7	4.38
2014	8	5.00
2015	6	3.72
2016	5	3.13
**JOURNAL**		
NeuroImage	57	35.6
Human Brain Mapping	26	16.3
Brain	8	5.0
Journal of International Neuropsychological Society	8	5.0
Biological Psychiatry	7	4.4
Neuropsychologia	7	4.4
Neurocase	6	3.8
Psychiatry Investigation	6	3.8
Neuroscience	5	3.0
Brain & Language	4	2.5
Cognitive Brain Research	4	2.5
The Journal of Pain	4	2.5
Brain Research	3	1.8
PLoS ONE	3	1.8
Brain and Cognition	2	1.3
Cortex	2	1.3
Experimental Neurology	2	1.3
Neurobiology of Learning and Memory	2	1.3
Archives of General Psychiatry	1	0.6
Cerebral Cortex	1	0.6
Frontiers in Systems Neuroscience	1	0.6
The Journal of Neuroscience	1	0.6
TECHNIQUE USED^**^		
SEM	135	84.4
Path Analysis	14	8.8
Unified SEM	2	1.3
Extended unified SEM	9	5.5
**TYPE OF DESIGN**		
Box car one group	79	50.6
Box car two groups	43	27.6
Box car more than two groups	6	3.8
Simple event related	11	7.1
Complex event related	6	3.8
Conjunction design	1	0.7
Resting state	10	6.4
**STRATEGY OF COMPARISONS**		
Between Subjects	19	11.9
Between Groups	27	16.9
Between Tasks	84	52.4
Factorial Task and groups	30	18.8
**TYPE OF POPULATION STUDIED**		
Healthy/Normal	87	54.4
Clinical	23	14.4
Both	42	26.3
Simulation study^*^	8	5.0
**KIND OF STUDY**		
Data driven	66	41.2
Hypothesis driven	63	39.4
Both	31	19.4
**NON-RECURSIVE EFFECTS**		
Yes	76	47.5
No	84	52.5
ESTIMATION TECHNIQUE^**^		
ML (Maximum Likelihood)	114	71.3
WLS (Weighted Least Squares)	7	4.4
Bootstrap	1	0.6
Others	1	0.6
No information	37	23.1
MULTINORMALITY ANALYSIS^**^		
Yes	18	11.3
No information	142	88.7
MATRIX ANALYZED^**^		
Correlation	22	13.8
Covariance	134	83.7
No information	4	2.5
CONDITIONS STUDIED^**^		
Well-conditioned	18	11.3
No information	142	88.7
***P*****-VALUE ASSOCIATED TO CHI SQUARE**		
Inferior to 0.10	37	42.0
Superior or equal to 0.10	51	58.0
DETERMINATION COEFFICIENT^**^		
Yes	4	2.5
No information	156	97.5
OTHER FIT INDEXES REPORTED^**^		
Yes	124	77.5
No	36	22.5

* *This category was eliminated in the posterior inferential analyses due to low frequency*.

** *These variables were eliminated in the inferential posterior analysis due to asymmetrical and low informative observed distributions*.

**Table 4:** Statistical descriptive of quantitative variables.

**Variables**	**Number of models**	**Mean**	**Standard deviation**	**Standard error**	**Range**
Total sample size	158	25.01	36.655	2.916	1–336
Clinical sample size	65	17.38	12.985	1.541	2–46
Healthy sample size	135	20.13	38.616	3.324	1–336
Number of brain areas analyzed	160	6.72	3.499	0.277	3–18
Number of defined paths	145	10.65	9.311	0.773	1–57
Chi square value	85	50.18	162.872	17.666	0.05–795.58
*p*-value of chi square	88	0.336	0.361	0.039	0–0.999
*R^2^*-value	160	0.341	0.212	0.020	0.11–0.84

As can be observed in the above tables, the SEMs studied present some interesting characteristics in accordance with the Carp structure (2012). If we try to define a prototypical SEM model for the study of functional connectivity, it would appear in a paper published between 2009 and 2012 (77 models, representing 48.1% of the total studied), published in the journals NeuroImage or Human Brain Mapping (83 models, i.e., 51.9%), based on the type-III SEM general model (135 models, i.e., 84.4%), extracting the ROIs after a cognitive paradigm based on a block design (Box-Car) or on some of its modifications (128 models, accounting for 80.0%), and which rarely use complete factorial designs (only 30 models, 18.8% of the total). It would be a SEM generated with real samples (only 5% simulate data), and the model specification would as likely come from statistical significances (66 models used a Data-Driven strategy, i.e., 41.3%) as from hypotheses with a neurobiological basis (63 models used that strategy, i.e., 39.4%). The parameter estimations would have been conducted through ML (114 models, 71.3%), and we would have no information on whether the study complies with the condition of application of multinormality for SEM (142 models offer no data on this aspect, i.e., 88.8%). The initial solution in the estimation process would stem from a matrix (*S*) of second-order centered moments. We would have no data on whether the study complies with the conditions of application of SEM (for example, range or order) given that 142 models offer no data on this (88.8% of the total analyzed). Additionally, from Table 4 we could infer that the general sample would comprise about 25 subjects (± 36.65), which implies a very wide variability in the sample sizes used and which, in mean values, is slightly above Friston's recommendation (Friston, 2012)—around 16 subjects per group analyzed, disregarding some recent clarifications on this topic (Lindquist et al., 2013). Moreover, the specific model would include around 6 or 7 ROIs (±3.499) and between 10 and 11 effects would have been specified (±9.311). Both results indicate a very high variability, which implies dispersion in the formulations and, given that we can expect many more effects specified than the number of ROIs involved, recursive effects would come as no surprise. As regards the fit data, we would have the usual indexes of fit in SEM, like the Goodness of Fit Index (GFI), or the Adjusted Goodness of Fit Index (AGFI), or the Comparative Fit Index (CFI) or the Akaike Criteria (AIC), or the Bayesian Criteria (BIC) among many others (124 models offer this type of information, 77.5%). Likewise, we would obtain some evidence of the global fit since the *p*-value associated with χ^2^ would be >0.10 (51 models offer this fit, which means 58% of the 88 models with this information and represent 31,9% of the totals of the analyzed models); although it would not be unusual to have SEM models with *p*-values associated with χ^2^ below the usual criterion, as this is perfectly comparable to the majority of SEM published in other fields. It is unusual to show the percentage of variance explained (*R*^2^) by the model (only 2.5% of the models analyzed offer this information) and the χ^2^-values offered would have a mean value of 50.18 (±162.87), which shows a very high variability and low consistency. From the above, only four of the total of 160 models include the explicit value of *R*^2^ associated with each model; in the other 156 models the value of *R*^2^ was estimated from the procedure discussed above.

Finally, in this case, the estimates of *R*^2^ in each model can be considered independent of the sample size used since the sample estimates of *R* (*S*) and that of Σ do not depend on this value. In the same way, we can assume that the individual estimate of *R*^2^ is equal to the common weighted estimate since the estimation procedure was applied equally in all the models, thus guaranteeing the homogeneity of *R*^2^ in all the models studied.

### Effects of the moderator variables on the *R^2^*-value

For this section, we decided to use the estimations derived from the fit of a General Linear Model, as is usual in classical meta-analyses, in order to establish the differential effects between each of the categories of the moderating variables used, or to estimate the correlations between the distributions of the quantitative moderating variables. In both cases, the corresponding parameters were estimated through ANOVA for the categorical variables and through Linear Regression for the quantitative variables.

As is well-known, estimations of effect size in any meta-analysis are subject to several perverse effects which may generate bias in the process: in this case, the estimation of *R*^2^ in each of the models which did not report it (i.e., 154 of the 160 studied). Some of the usual problems are related to the different number of variables (ROIs) involved in each model and dissimilar sample sizes. In line with Cheung (2015), we approached these two issues based on the conceptions of the SEMs for the study of meta-analyses (Meta-Analysis Structural Equation Models, MASEM).

In this case, we applied the strategy described by Cheung (2015), using SEMs to estimate the homogeneity of effect sizes (*Q* index) and the percentage of variation attributable to the models analyzed (*I*^2^ index). To estimate both indicators, we used the following expressions:

$$\begin{array}{l} Q=k{\hat{\sigma }}_{{ẽ}_{i}}^{2}, \end{array}$$

where ${\hat{\sigma }}_{{ẽ}_{i}}^{2}=\;\overset{k}{\underset{i=1}{\sum }}\frac{{\left({ỹ}_{i}-\sqrt{w_{i}}\;{\hat{\beta }}_{F}\right)}^{2}}{k}\;$, which involves the estimation of the error variance ẽ_*i*_ in the *k* models, using (*k*) instead of (*k* − *1*) for a better adjustment to the demands of the maximum likelihood estimations (*ML*) and following a χ^2^ distribution. Along with this premise, the estimation of *I*^2^ was conducted with the simple expression

$$\begin{array}{l} I^{2}=1-\;\frac{k-1}{Q} \end{array}$$

Both values showed the heterogeneity of the effect sizes (*Q*^2^ = 1032.46; *df* = 160; *p* < 0.001) and the variation can be explained by the effects between models rather than by intra variability (*I*^2^ = 0.846). In the light of these results we estimated the effects corresponding to each moderator variable by means of Mplus (MASEM). Tables 5, 6 summarize the information from both analyses with specification of the β_*ij*_ parameters (impact of each variable on *R*^2^) for each variable or category, depending on the case.

**Table 5:** Effects in value *R*^2^ for qualitative moderators.

**Variables**	**k**	***β_*ij*_***	**SE_β_**	***p*-value**	**95% confidence interval of** ***β_*****ij*****_***		***$\eta_{partial}^{2}$***
					**Lower limit**	**Upper Limit**	
**TYPE OF DESIGN**							
Box car one group	56	0.824	0.084	<0.001	0.657	0.991	0.462
Box car more than one group	34	1.100	0.108	<0.001	0.885	1.314	0.482
Event related	15	0.965	0.163	<0.001	0.642	1.288	0.240
Resting state	10	0.589	0.200	0.004	0.193	0.985	0.073
**COMPARISON**							
Subjects	17	0.594	0.153	<0.001	0.292	0.897	0.116
Groups	21	0.925	0.137	<0.001	0.653	1.197	0.283
Task	61	0.907	0.081	<0.001	0.748	1.067	0.524
Task and groups	20	1.049	0.141	<0.001	0.770	1.327	0.325
**TYPE OF POPULATION STUDIED**							
Healthy/Normal	62	0.844	0.083	<0.001	0.680	1.009	0.488
Clinical	17	0.757	0.159	<0.001	0.442	1.072	0.174
Both	32	1.050	0.116	<0.001	0.820	1.280	0.432
**KIND OF STUDY**							
Data driven	50	0.866	0.089	<0.001	0.689	1.043	0.448
Hypothesis driven	46	0.810	0.093	<0.001	0.626	0.995	0.395
Both	23	1.098	0.132	<0.001	0.837	1.359	0.375
**RECURSIVE EFFECTS**							
Yes	64	0.844	0.080	<0.001	0.687	1.002	0.491
No	55	0.942	0.086	<0.001	0.772	1.112	0.508
***P*****–VALUE ASSOCIATED TO CHI SQUARE**							
Inferior to 0.10	27	0.949	0.131	<0.001	0.687	1.212	0.445
Superior or equal to 0.10	40	0.961	0.108	<0.001	0.745	1.177	0.549

*k, number of models for each category; β, estimation parameter for each effect; SE_β_, Standard Error of the parameter; p, p-value associated to the parameter significance; $\eta_{\text{partial}}^{\text{2}}$, β parameter effect size*.

**Table 6:** Effects on *R*^2^ values for quantitative moderators.

**Variables**	**k**	***β_*ij*_***	**SE_β_**	***p***	***r^2^***	**95% confidence interval of** ***β_*****ij*****_***	
						**Lower limit**	**Upper limit**
Total sample size	158	−0.002	0.002	0.405	0.006	−0.005	0.002
Clinical sample size	65	−0.010	0.008	0.226	0.028	−0.025	0.006
Healthy sample size	135	−0.002	0.002	0.410	0.007	−0.006	0.002
Number of brain areas analyzed	160	−0.006	0.016	0.710	0.001	−0.037	0.025
Number of defined paths	145	0.021	0.006	0.001	0.106	0.009	0.032
Chi square value	85	−0.001	0.000	0.050	0.061	−0.002	0.000
*p*-value of chi square	88	0.081	0.235	0.733	0.002	−0.388	0.549

*k, number of models; β, estimation parameter; SE_β_, Standard Error of the parameter; p, p-value associated to the parameter significance; r^2^, Effect size*.

When studying the effect of the moderating variables on the value of *R*^2^ for each structural model analyzed, the first point to note is the larger size of the effect generally observed in the different values of the qualitative variables (Table 5). Additionally, the type of design with the greatest effect is Box Car, whether it is one group (β = 0.824; *p* < 0.001, η^2^ = 0.462) or more than one group (β = 1.100; *p* < 0.001, η^2^ = 0.482). The Resting State design category is also statistically significant, although with a very low effect (β = 0.589; *p* = 0.004, η^2^ = 0.073). The models in which the tasks are compared present the greatest effect and their intensity is high (β = 0.907; *p* < 0.001, η^2^ = 0.524). As regards the type of population studied, in studies based on healthy subjects that present the greatest effect (β = 0.844; *p* = < 0.001, η^2^ = 0.488), although the effect is similar to that found in studies based on both normal and clinical populations (β = 1.050; *p* = < 0.001, η^2^ = 0.432). The models that do not account for recursive effects present the greatest effect, and with a high-intensity effect size in this case (β = 0.942; *p* < 0.001, η^2^ = 0.508). Finally, as regards the *p*-value associated with the chi-square value, the models presenting a *p* > 0.10 yield a high-intensity effect size measurement (β = 0.961; *p* < 0.001, η^2^ = 0.549).

With regard to the quantitative variables of the value of *R*^2^, the significant effect is the number of paths established in the model (β = 0.021; *p* = 0.001, *r*^2^ = 0.106). Though a low-intensity effect, it is important to remember that the association with the *R*^2^ of the SEM model is positive; this suggests that a complex effect could occur according to which, with more complex models, we would obtain some more explained variance, i.e., higher in statistical terms, but not necessarily yielding a higher number of significant parameters. This effect, widely described in other areas, is equally important here with regard to the idea of complexity associated with a possible network of functional connectivity.

## Conclusions

Firstly, it is important to mention a few details about the use of SEMs in the field of functional or effective connectivity. An interesting piece of information is the rapid development in studies of this type, since many functional connectivity models have appeared in recent years in different fields and tasks. Therefore, as a consequence of the above comment, and most importantly, the researchers seem to have found a good statistical tool in SEM models for the development of some concepts, models, and answers in Computational Cognitive Neuroscience. Nonetheless, it should be noted that 64.39% of the models analyzed here were published between 2009 and 2016. This suggests a greater increase in recent years. This increase coincides with a clear general increase on brain connectivity with fMRI, which obviously implies an increase in the use of analysis techniques related to estimates of connectivity according with the results of Welvaert and Rosseel (2014).

Likewise, a certain concentration exists in the media; it is striking that 51.9% of the papers were published in the Journal of NeuroImage and Human Brain Mapping, in accordance with some of the precepts of Bradford's Law of bibliometrical studies. This major concentration of publications has a bearing on the dissemination of results. From a more specific viewpoint, note that the classical SEM model is the most widely used; 84.4% of the models analyzed use the classical LISREL model. Therefore, there is still room for the development of alternatives like euSEM, uSEM, ESEM, or Bayesian approaches.

From a more methodological perspective, we should also point out that the data analyzed in the models were generated from block designs in 82.0% of the cases, with a relative presence of event-related designs. This could be interpreted as evidence of the difficulties of event-related designs with the type of signal analyzed here (*f* MRI), which led researchers to set forth simpler, more secure designs when registering and treating the signal.

Yet another important finding is the fact that only 5% of the models were generated with simulated data, so the choice of data simulation to estimate functional connectivity models is an issue that should be explored in greater depth. We are not saying that this is the most adequate choice when facing real data, but we *should* consider it as a choice for the behavior of connectivity models, as a statistical model. Likewise, this involves a willingness to work with real samples, which points to an evident need for connectivity model results that contribute to the development of applied knowledge.

Lastly, in this rather instrumental section, note that the most widely used estimation technique is ML (Maximum Likelihood, used in 71.3% of the models). Few papers pay attention to the statistical assumptions of SEM models (for example, 88.8% of the models do not report on the distribution of the values of each ROI). SEM models usually employ between 6 and 7 ROIs (*M* = 6.72 and *SD* = 3.499) in their formulation and establish between 10 and 11 (*M* = 10.65 and *SD* = 9.311) effects as free parameters, according with descriptive of the table number 4. They are, therefore, relatively small models in relation to the number of variables (ROIs) incorporating a limited number of effects. Probably, this limited conception of SEMs may compromise the chances of generalizing the results, even if applied to real samples, given that only models with few brain areas are analyzed—a situation that differs markedly from the evident complexity of real brain connectivity structures.

As we value the results obtained in this paper on the relationship between the moderating variables and the *R*^2^-values of the structural models, we would like to highlight some effects for each of the groups of moderating variables.

Box-Car designs offer higher estimations in the *R*^2^ than other types of designs (η^2^ = 0.462 or η ^2^ = 482), with somewhat higher effects than the block designs with more than one group. The effect in *R*^2^ is less significant in the case of event-related (η^2^ = 0.240) and even less in the resting state procedure (η^2^ = 0.073). This result could be interpreted as suggesting that the identification of a significant response on fMRI signal to a stimulus is a better way of estimating more interesting correlations from the SEM estimation parameter point of view than the situation in which the signal only includes basal values. Therefore, by using ML as the estimation technique, block designs—of one group or more—are related to higher *R*^2^-values.

In the case of the statistical contrast incorporated in base designs, it seems advisable for the analyses at the first level to use comparison between tasks than between groups. Comparing tasks as first level analysis generates higher *R*^2^-values (η^2^ = 0.524) than the other usual comparisons in fMRI designs (between groups, subjects or interaction task by groups).

With regard to the characteristics of the samples, it seems that, for the *R*^2^-values, the samples of healthy subjects offer better estimations (η^2^ = 0.488) than the rest, with the exception of designs with healthy and control groups. Be that as it may, in this case there is an evident relation to the highest values of the parameters with designs that allow a certain tendency to use samples with healthy subjects for the extraction of ROIs and, in a one- or two-group design strategy, a comparison between activations in the face of different tasks.

If we look at the model generation strategy, it seems that data-driven models offer better estimations in *R*^2^-values (η^2^ = 0.448). Therefore, the data-driven resource seems to offer better estimations, which matches what we know about circular structures in this type of studies (Kriegeskorte et al., 2009). Consequently, if the appropriate measures are not taken when defining orthogonal contrasts in the first phase of analysis, we may have oversignificance and, therefore, an obvious circularity in the final adjusted model.

Likewise, models with non-recursive effects seem to offer higher estimations (η^2^ = 0.508) of the structural parameters and may be slightly more desirable structures than models with recursive effects. This may also be linked to the limited ability of SEMs to represent very complex structures, an issue which has been the object of several papers; the limitations are reproduced here when applied to the estimation of functional connectivity (Cheung, 2013).

However, as regards the models' degree of significance a clear effect exists on the *R*^2^-value of the structural models. The effect size is patently greater in the models with a good fit, that is, with degrees of significance greater than or equal to 0.10 (η^2^ = 0.549). In this case, the paper by Wua and Kwokb (2012) shows very similar effects in complex models related to the multilevel approach.

Interesting findings have also been reported for other quantitative moderating variables. There is a mild effect associated with the number of paths defined in model in relation to the value of *R*^2^ (*r*^2^ = 0.106) with a positive relationship between the number of paths defined and the total value of the explained variance. However, this effect has a mild impact. In this area, no other statistical effects deserve further comment.

In summary, the analyses presented here indicate that if the aim is to establish a SEM to study functional connectivity derived from a work with *f* MRI signal, the likelihood of obtaining high structural parameters and, consequently, high *R*^2^-values can be maximized by the use of Box-Car designs in one or more groups rather than event-related or resting state procedures. Equally it seems better to use first level analysis comparing tasks or mixed designs (tasks by group) and designs with samples of healthy subjects and including the statistically significant effects of the first level analysis using the data-driven strategy. Moreover, it may be more interesting to specify non-recursive models in order to generate more complex models. This configuration, according to our data, is associated with better χ^2^ fit values (*p* > 0.10) and with higher values of explained variance (*R*^2^). Finally, the number of defined paths present a low contribution to the better results in SEMs estimation and adjust.

Evidently, this paper has some limitations that should be noted and which derive from the scheme we apply. For instance, we did not bear in mind the specific structures involved in each model, so we do not yet have information on the models' neuroanatomical plausibility. Likewise, we did not conduct a study of those neuroanatomical structures to assess the reiteration of structures involved in similar models. Nor did we bear in mind the characteristics of the cognitive functions used in the definition of the activations, so it is possible that specific tasks may offer more consistent *f* MRI activations and may therefore be associated with the fit of more complex, powerful models in statistical terms. This would hardly come as a surprise since enough evidence exists of associations of cognitive tasks, like motor tasks, with more statistically significant *f* MRI activations. Likewise, a clear limitation is the fact that we did not study all the parameters of the models analyzed, but focused solely on the *R*^2^-values instead. The study of all the parameters would have indicated whether their distribution presented some systematic bias that generates insufficient biased estimations; for example, by applying the BLUE (Best Linear Unbiased Estimator) precepts to SEMs fitted according to the study of ROIs with a statistically significant activation in cognitive paradigms assessed through a *f* MRI signal.

In spite of all of the above, we have found no previous papers on these matters and, therefore, we consider that our results and assessments may represent an initial guideline to the use of SEMs for the estimation of functional connectivity. Below we have set out a series of recommendations for applied researchers intending to use SEM models for the adjustment of functional connectivity models. Very briefly, we suggest the following:

- Pay attention to the statistical assumptions of the SEM models, assessing to what extent they are complied with and to what extent not doing so may affect the parameter estimation process. Special attention should be paid to the observed distribution of each ROI based on the values obtained through PCA or other techniques. Anomalous distributions yield aberrant estimations if ML is used with no added corrections.
- Clearly identify whether the estimations are standardized or not; if they are not, provide the standard error estimations for each parameter.
- Identify whether the estimation process is conducted based on a variance-covariance matrix (*S*), or a correlations matrix (*R*), given that the use of centered moments or centered and standardized moments involve rather different processes.
- For the whole SEM model analyzed, offer the values of global fit. Without them, it is impossible to conduct a correct analysis of viability. Therefore, the values of χ^2^, the degrees of freedom, the associated degree of significance, and a complete list of indexes of fit should become good praxis in the presentation of SEM models.
- Likewise, the use of the Coefficient of Determination (*R*^2^) should be incorporated and normalized to determine the model's degree of impact with regard to the explained variation of the ROIs included. It offers relevant information about the importance of the adjusted model and its true impact in statistical terms.
- Bear in mind that the value of the explained variance is not independent of the number of ROIs or of the number of paths defined. It seems that SEM models present some limitations with regard to the number of ROIs and effects.
- It seems advisable to use block designs to obtain activations in the first level of analysis. In the same type of design, the comparison between tasks seems to offer greater estimations than the comparison between groups.
- From the point of view of the statistical approach to functional connectivity, the SEM established from Data-Driven strategies may be preferable, despite the fact that Hypothesis-Driven models present a greater validity of content and may therefore be more realistic from a neuroanatomical viewpoint.
- In the latter case, the study of samples of healthy subjects is also related to higher values in the estimation process and, therefore, it is preferable to include a group of healthy subjects in the design to compare their *f* MRI activations to those of the clinical samples.
- Bear in mind that the structures that SEM models can represent must be simple, without a very high number of ROIs, and that the definition of recursive and non-recursive effects is not independent of the results we will obtain. The simplest structures seem to offer the greatest estimations.

## Author contributions

All authors listed have made a substantial, direct and intellectual contribution to the work, and approved it for publication.

### Conflict of interest statement

The authors declare that the research was conducted in the absence of any commercial or financial relationships that could be construed as a potential conflict of interest.
