# Supplementary material for: Meta-Analysis of the Structural Equation Models' Parameters for the Estimation of Brain Connectivity with fMRI
Source: Front Behav Neurosci. 2018 Feb 15;12:19. doi: 10.3389/fnbeh.2018.00019 (PMC5818469; doi:10.3389/fnbeh.2018.00019)
Supplement: Supplementary file 1 [file DataSheet1.docx]

**References**

Berry, W.D. (1984). *Nonrecursive Causal Models*. Beverly Hills: Sage Pub. Inc.

Bollen, K.A., & Scott-Long, J. (1993). *Testing Structural Equation Models*. New York: Sage.

Brown, T.A. (2006). *Confirmatory Factor Analysis for Applied Research*. New York. Guilford Press.

Bringmann, L. F., Scholte, H. S., & Waldorp, L. J. (2013). Matching structural, effective, and functional connectivity: a comparison between structural equation modeling and ancestral graphs. *Brain Connectivity*, *3*(4), 375-385.

Byrne, B.M. (2010). *Structural Equation Modeling with AMOS*. New York: Taylor & Francis.

Carp, J. (2012). The secret lives of experiments: methods reporting in the fMRI literature. *Neuroimage*, *63*(1), 289-300.

Chen, G., Glen, D. R., Saad, Z. S., Hamilton, J. P., Thomason, M. E., Gotlib, I. H., et al. (2011). Vector autoregression, structural equation modeling, and their synthesis in neuroimaging data analysis. *Computers in Biology and Medicine*, *41*, 1142–1155.

Cheung, M.L.W. (2015). *Meta-Analysis. A Structural Equation Modeling Approach*. Chuchester: John Wiley & Sons, Ltd.

Cheung, M.W. (2013). Multivariate Meta-Analysis as Structural Equation Models. *Structural Equation Modeling*, *20*(3), 429-454.

De Marco, G., Vrignaud, P., Destrieux, Ch., De Marco, D., Testelin, S., Devauchelle B., et al. (2009). Principle of structural equation modeling for exploring functional interactivity within a putative network of interconnected brain areas. *Magnetic Resonance Imaging*, *27*, 1–12.

Deshpande, G., Hu, X. Investigating Effective Brain Connectivity from fMRI Data: Past Findings and Current Issues with Reference to Granger Causality Analysis. (2012). *Brain Connectivity*, *2*, 235-245.

Everitt, B., & Hothorn, T. (2011). *An introduction to applied multivariate analysis with R*. New York: Springer.

Farràs-Permanyer, L., Guàrdia-Olmos, J., & Peró-Cebollero, M. (2015). Mild cognitive impairment and fMRI studies of brain functional connectivity: the state of the art. *Frontiers in Psychology*, 6.

Friston, K.J. (2012). Ten ironic rules for non-statistical reviewers. *NeuroImage*, *61*, 1300-1310.

Gates, K.M. & Molenaar, P.C.M. (2012). Group search algorithm recovers effective connectivity maps for individuals in homogeneous and heterogeneous samples. *NeuroImage*, *63*, 310–319.

Jöreskog, K.G.,Sörbom, D. (1979). *Advances if factor analysis and structural equation models*. Massachusetts: Abt. Books.

Jöreskog, K.G.,Sörbom, D. (1984). *Lisrel VI. Analysis of linear structural relationships by maximum likelihood, instrumental variables and least square methods*. New York: Scientific Software Inc.

Kline, R.B. (2011). *Principles and Practice of Structural Equation Modeling*. New York: Guilford.

Kriegeskorte, N., Lindquist, M. A., Nichols, T. E., Poldrack, R. A., & Vul, E. (2010). Everything you never wanted to know about circular analysis, but were afraid to ask. *Journal of Cerebral Blood Flow & Metabolism*, *30*(9), 1551-1557.

Kriegeskorte, N.; Simmons, W.K., Bellgowan, P., Baker, Ch.I. (2009). Circular analysis in systems neuroscience – the dangers of double dipping. *Nature Neuroscience*, *12*(5), 535–540. doi:10.1038/nn.2303.

Lange, N.D., Davelaar, E.J., Thomas, R.P. (2013). Data acquisition dynamics and hypothesis generation. *Cognitive Systems Research*, *24*, 9-17.

Lindquist, M.A., Caffo, B., Crainiceanu, C. (2013). Ironing out the statistical wrinkles in “ten ironic rules”. *NeuroImage*, *81*, 499-502.

MacIntosh, A.R. (1999). Mapping Cognition to the Brain Through Neural Interactions. *Memory*, *7*, 523-548.

McIntosh, A.R., Gonzalez-Lima, F. (1991). Structural modeling of functional neural pathways mapped with 2-deoxyglucose: effects of acoustic startle habituation on the auditory system. *Brain Research*, *547*, 295-302.

McIntosh, A.R., Gonzalez-Lima, F. (1992). The application of structural modeling to metabolic mapping of functional neural systems. *Advances in Metabolic Mapping Techniques for Brain Imaging of Behavioral and Learning Functions*, *68*, 219-255.

McIntosh, A.R., Gonzalez-Lima, F. (1994). Structural Equation Modeling and Its Application to Network Analysis in Functional Brain Imaging. *Human Brain Mapping*, *2*, 2-22.

Moreira, P.S.; Sotiropoulos, I.; Silva, J.; Takashima, A.; Sousa, N.; Leite-Almeida, H.; & Costa, P.S. (2016). The Advantages of Structural Equation Modeling to Address the Complexity of Spatial Reference Learning. *Frontiers in Behaviuoral Neuroscience*, 10.

Murray, A.D., Staff, R.T., McNeil, C.J., Salarirad, S., Starr, J.M., Deary, I.J., et al. Brain lesions, hypertension and cognitive ageing in the 1921and 1936 Aberdeen birth cohorts. Age, 2012; 34, 451-459. doi 10.1007/s11357-011-9233-5.

Penke, L.,Deary, I.J. (2010). Some guidelines for structural equation modeling in Cognitive neuroscience: The case of Charlton et al.’s study on white matter integrity and cognitive ageing. *Neurobiology of Aging*, *31*, 1656-1660.

Penny, W.D., Stephan, K.E., Mechelli, A.,Friston, K.J. (2004). Modelling functional integration: a comparison of structural equation and dynamic causal models. *NeuroImage*, *23*, 264-274.

Price, L.R. (2012). Small Sample Properties of Bayesian Multivariate Autoregressive Time Series Models. *Structural Equation Modeling*, *19*, 51–64.

Raykov, T., & Marcoulides, G.A. (2006). *A First Course in Structural Equation Modeling*. New York: Taylor & Francis.

Redondo, S., Sánchez, J., Garrido, V. (2002). Los programas psicológicos con delincuentes y su efectividad: La situación europea. *Psicothema*, *14*, 164-173.

Sato, J.R.; Dean, Ph.J.A.; & Vieira, G. (2014). *Methods for Connectivity Analysis in fMRI*. In Methods in Brain Connectivity Inference through Multivariate Time Series Analysis, (197 – 222). New York: CRC Press-Taylor & Francis Group.

Sawyer, K.S., Corsentino, E., Sachs, N., Steffens, D.C. (2012). Depression, Hippocampal Volume Changes, and Cognitive Decline in a Clinical Sample of Older Depressed Outpatients and Non-depressed Controls. *Aging Mental Health*, *16*, 753-762. doi:10.1080/13607863.2012.678478.

Schlösser, R.G.M., Wagner, G., Sauer, H. (2006). Assessing the working memory network: studies with functional magnetic resonance imaging and structural equation modeling. *Neuroscience* , *139*, 91-103.

Schwarzer, G. (2013). Meta-Analysis with R. Document donwload from http://mirror.ufs.ac.za/cran/web/packages/meta/meta.pdf on july, 30, 2016.

Taylor, J.G., Krause, B., Shah, N.J., Horwitz, B., Mueller-Gaertner, H.W. (2010). On the Relation Between Brain Images and Brain Neural Networks. *Human Brain Mapping*, *9*, 165-182.

Vesterinen, H.M., Sena, E.S., Egan, K.J., Hirst, T.C., Churolov, L., Currie, G.L., … (2014). Meta-analysis of data from animal studies: A practical guide. *Journal of Neuroscience Methods*, *221*, 92 – 102.

Voineskos, A.N., Rajji, T.K., Lobaugh, N.J., Miranda, D., Shenton, M.E., Kennedy, J.L., … (2012). Age-related decline in white matter tract integrity and cognitive performance: A DTI tractography and structural equation modeling study. *Neurobiology of Aging*, *33*, 21–34.

Vul, E., Harris, C., Winkielman, P., & Pashler, H. (2009). Puzzlingly high correlations in fMRI studies of emotion, personality, and social cognition. *Perspectives on Psychological Science*, *4*(3), 274-290.

Welvaert, M. & Rossell, Y. (2014). A Review of fMRI Simulation Studies. *Plos One*, Vol. 9, 7, e101953. doi:10.1371/journal.pone.0101953.

Wua. J.Y. & Kwokb, O.M. (2012). Using SEM to analyze complex survey data: A comparison between design-based single-level and model-based multilevel approaches. *Structural Equation Modeling*, *19*(1), 16-35.

Yang, Y., Joshib, A.A., Joshia, S.H., Bakerc, L.A., Narr, K.L., Raine, A., … (2012). Genetic and environmental influences on cortical thickness among 14-year-old twins. *NeuroReport*, *23*, 702-706.

**Papers included in the meta-analysis study**

Alonso-Montes, C.; Diez, I.; Remaki, L.; Escudero, I.; Mateos, B.; Rosseel, Y.; Marinazzo, D.; Stramaglia, S.; & Cortes, J. (2015). Lagged and instantaneous dynamical influences related to brain structural connectivity. *Frontiers in Psychology*, 6.

Barbalat, G., Chambon, V., Domenech, P. J. D., Ody, C., Koechlin, E., Franck, N., et al. (2011). Impaired hierarchical control within the lateral prefrontal cortex in schizophrenia. *Biological Psychiatry*, *70*, 73-80.

Beltz, A. M., & Molenaar, P. (2015). A posteriori model validation for the temporal order of directed functional connectivity maps. *Frontiers in Neuroscience*, 9.

Beltz, A. M., Beekman, C., Molenaar, P. C., & Buss, K. A. (2013). Mapping temporal dynamics in social interactions with unified structural equation modeling: A description and demonstration revealing time-dependent sex differences in play behavior. *Applied Developmental Science*, *17*(3), 152-168.

Beltz, A. M., Gates, K. M., Engels, A. S., Molenaar, P. C., Pulido, C., Turrisi, R., ... & Wilson, S. J. (2013). Changes in alcohol-related brain networks across the first year of college: a prospective pilot study using fMRI effective connectivity mapping. *Addictive Behaviors*, *38*(4), 2052-2059.

Bianchi, A.M., Marchetta, E., Tana, M.G., Tettamanti, M., Rizzo, G. (2012). Frequency-based approach to the study of semantic brain networks connectivity. *Journal of Neuroscience Methods*, *212*, 181- 189.

Boyer, L.; Testart, J.; Michel, P.; Richieri, R.; Faget-Agius, C.; Vanoye, V.; Auquier, P.; Lancon, Ch.; & Guedj, E. (2014). Neurophysiological correlates of metabolicsyndrome and cognitive impairment inschizophrenia: A structural equation modeling approach. *Psychoneuroendocrinology*, *50*, 95 – 105.

Craggs, J. G., Price, D. D., Verne, G. N., Perlstein, W. M., Robinson, M. M. (2007). Functional brain interactions that serve cognitive–affective processing during pain and placebo analgesia. *NeuroImage*, *38*, 720-729.

Craggs, J.G., Staud, R., Robinson, M.E., Perlstein, W.M., Price, D.D. (2012). Effective connectivity among brain regions associated with slow temporal summation of C-Fiber-Evoked pain in fibromyalgia patients and healthy controls. *The Journal of Pain*, *13*(4), 390-400.

Dick, A. S., Solodkin, A., Small, S. L. (2010). Neural development of networks for audiovisual speech comprehension. *Brain & Language*, *114*, 101-114.

Erickson, K. I., Ho, M-H. R., Colcombe, S. J., Kramer, A. F. (2005). A structural equation modeling analysis of attentional control: an event-related fMRI study. *Cognitive Brain Research*, *22*, 349-357.

Fehlings, M.G. (2016). Changes in Pain Processing in the Spinal Cord and Brainstem after Spinal Cord Injury Characterized by Functional Magnetic Resonance Imaging. *Journal of Neurotrauma*, *33*(15), 1450 - 1460.

Fino, E., Melogno, S., Iliceto, P., D’Aliesio, S., Pinto, M. A., Candilera, G., & Sabatello, U. (2014). Executive functions, impulsivity, and inhibitory control in adolescents: A structural equation model. *Advances in Cognitive Psychology*, *10*(2), 32.

Flagmeier, S. G., Ray, K. L., Parkinson, A. L., Li, K., Vargas, R., Price, L. R., ... & Robin, D. A. (2014). The neural changes in connectivity of the voice network during voice pitch perturbation. *Brain and Language*, *132*, 7-13.

Fu, C. H. Y., McIntosh, A. R., Kim, J., Chau, W., Bullmore, E. T., Williams, S. C. R., et al. Modulation of effective connectivity by cognitive demand in phonological verbal fluency. NeuroImage, 2006; 30, 266-271.

Gates, K. M., Molenaar, P. C. M., Hillary, F. G., Ram, N., Rovine, M. J. (2010). Automatic search for fMRI connectivity mapping: An alternative to Granger causality testing using formal equivalences among SEM path modeling, VAR, and unified SEM. *NeuroImage*, *50*, 1118-1125.

Gates, K. M., Molenaar, P. C. M., Hillary, F. G., Slobounov, S. (2011). Extended unified SEM approach for modeling event-related fMRI data. *NeuroImage*, *54*, 1151-1158.

Gavrilescu, M., Stuart, G. W., Waites, A., Jackson G., Svalbe, I. D., Egan, G. F. (2004). Changes in effective connectivity models in the presence of task-correlated motion: an fMRI study. *Human Brain Mapping*, *21*, 49-63.

Golfinopoulos, E., Tourville, J. A., Bohland, J. W., Ghosh, S. S., Nieto-Castanon, A., Guenther, F. H. (2011). fMRI investigation of unexpected somatosensory feedback perturbation during speech. *NeuroImage*, *55*, 1324-1338.

Gonçalves, M. S., Hall, D. A. (2003). Connectivity analysis with structural equation modelling: an example of the effects of voxel selection. *NeuroImage*, *20*, 1455-1467.

Gonçalves, M. S., Hall, D. A., Johnsrude, I. S., Haggard, M. P. (2001). Can meaningful effective connectivities be obtained between auditory cortical regions? *NeuroImage*, *14*, 1353-1360.

Guardia-Olmos, J.; Pero-Cebollero, M.; Zarabozo-Hurtado, D.; González-Garrido, A.; & Gudayol-Ferre, E. (2015). Effective connectivity of visual word recognition and homophone orthographic errors. *Frontiers in Psychology*, 6.

Haase, L., Wang, M., Green, E., Murphy, C. (2011). Functional connectivity during recognition memory in individuals genetically at risk for Alzheimer’s Disease. *Human Brain Mapping*, *34*, 530-542.

Hill, B. D., Foster, J. D., Elliott, E. M., Shelton, J. T., McCain, J., & Gouvier, W. D. (2013). Need for cognition is related to higher general intelligence, fluid intelligence, and crystallized intelligence, but not working memory. *Journal of Research in Personality*, *47*(1), 22-25.

Hillary, F. G., Medaglia, J. D., Gates, K., Molenaar, P. C., Slocomb, J., Peechatka, A., et al. (2011). Examining working memory task acquisition in a disrupted neural network. Brain, *134*, 1555-1570.

Horowitz-Kraus, T., Vannest, J. J., Gozdas, E., & Holland, S. K. (2014). Greater utilization of neural-circuits related to executive functions is associated with better reading: a longitudinal fMRI study using the verb generation task. *Frontiers in Human Neuroscience*, *8*, Article 447.

Inman, C. S., James, G. A., Hamann, S., Rajendra, J. K., Pagnoni, G., Butler, A. J. (2012). Altered resting-state effective connectivity of fronto-parietal motor control systems on the primary motor network following stroke. *NeuroImage*, *59*, 227-237.

Jackson, M.C., Morgan, H.M., Shapiro, K.L., Mohr, H., Linden, D.E.J. (2011). Strategic resource allocation in the human brain supports cognitive coordination of object and spatial working memory. *Human Brain Mapping*, *32*, 1330-1348.

James, G. A., Kelley; M. E., Craddock; R. C., Holtzheimer, P. E., Dunlop, B. W., Nemeroff, C. B., (2009). Exploratory structural equation modeling of resting-state fMRI: Applicability of group models to individual subjects. *NeuroImage*, *45*, 778-787.

Karunanayaka, P. R., Holland, S. K., Schmithorst, V. J., Solodkin, A., Chen, E. E., Szaflarski, J. P., (2007). Age-related connectivity changes in fMRI data from children listening to stories. *NeuroImage*, *34*, 349-360.

Karunanayaka, P., Eslinger, P. J., Wang, J. L., Weitekamp, C. W., Molitoris, S., Gates, K. M., ... & Yang, Q. X. (2014). Networks involved in olfaction and their dynamics using independent component analysis and unified structural equation modeling. *Human Brain Mapping*, *35*(5), 2055-2072.

Karunanayaka, P., Schmithors, V. J., Vannest, J., Szaflarski, J. P., Plante, E., Holland, S. K. (2011). A linear structural equation model for covert verb generation based on independent component analysis of fMRI data from children and adolescents. *Frontiers in Systems Neuroscience*, *5*, 1–15.

Kim, J., Horwitz, B. (2009). How well does structural equation modeling reveal abnormal brain anatomical connections? An fMRI simulation study. *NeuroImage*, *45*, 1190-1198.

Kim, J., Zhu, W., Chang, L., Bentler, P. M., Ernst, T. (2007). Unified Structural Equation Modeling approach for the analysis of multisubject, multivariate functional MRI data. *Human Brain Mapping*, *28*, 85-93.

Kim, J.S., Jung, W.J., Kang, D.H., Park, J.I., Jang, J.H., Choi, J.S., et al. (2012). Changes in effective connectivity according to working memory load: an fMRI study of face and location working memory tasks. *Psychiatry Investigation*, *9*, 283-292.

Kiyama, S.; Kunimi, M.; Iidaka, T.; Nakai, T. (2014). Distant functional connectivity for bimanual finger coordination declines with aging: an fMRI and SEM exploration. *Frontiers in Human Neuroscience*, 8.

Kondo, H., Osaka, N., Osaka, M. (2004). Cooperation of the anterior cingulate cortex and dorsolateral prefrontal cortex for attention shifting. *NeuroImage*, *23*, 670- 679.

Levy; J., Pernet, C., Treserras, S., Boulanouar, K., Aubry, F., Démonet,J-F., et al. (2009). Testing for the dual-route cascade reading model in the brain: an fMRI effective connectivity account of an efficient reading style. *PLoS ONE*, *8*,1-13.

Li, K.; Laird, A.R.; Price, L.R.; McKay, D.R.; Blangero, J.; Glahn, D.C.; & Fox, P.T. (2016). Progressive Bidirectional Age-Related Changes in Default Mode Network Effective Connectivity across Six Decades. *Frontiers in Aging Neuroscience*, 8.

Lin, F-H., Agnew, J. A., Belliveau, J. W., Zeffiro, T. A. (2009). Functional and effective connectivity of visuomotor control systems demonstrated using generalized partial least squares and structural equation modeling. *Human Brain Mapping*, 30, 2232-2251.

Lu, C., Chen, C., Ning, N., Ding, G., Guo, T., Peng, D., et al. (2010). The neural substrates for atypical planning and execution of word production in stuttering. *Experimental Neurology*, *221*, 146-156.

Lu, C., Ning, N., Peng, D., Ding, G., Yang, K. Ll. Y., Lin, C. (2009). The role of large-scale neural interactions for developmental stuttering. *Neuroscience*, *161*, 1008-1026.

Lu, C., Peng, D., Chen, C., Ning, N., Ding, G., Li, K., et al. (2010). Altered effective connectivity and anomalous anatomy in the basal ganglia-thalamocortical circuit of stuttering speakers. *Cortex*, *46*, 49-67.

Ma, L., Wang, B., Narayana, S., Hazeltine, E., Chen, X., Robin, D. A., et al. (2010). Changes in regional activity are accompanied with changes in inter-regional connectivity during 4 weeks motor learning. *Brain Research*, *1318*, 64-76.

Mark, A., Chevillet, M.A., Jiang, X., Rauschecker, J.P., Riesenhuber, M. (2013). Automatic Phoneme Category Selectivity in the Dorsal Auditory Stream. *The Journal of Neuroscience*, *33*(12), 5208-5215.

Marques, P., Moreira, P., Magalhães, R., Costa, P., Santos, N., Zihl, J., ... & Sousa, N. (2016). The functional connectome of cognitive reserve. *Human Brain Mapping*, *37*(9), 3310-3322.

Marrelec, G., Kim, J., Doyon, J., Horwitz, B. (2009). Large-scale neural model validation of partial correlation analysis for effective connectivity investigation in functional MRI. *Human Brain Mapping*, *30*, 941-950

McCormick, C., Moscovitch, M., Protzner, A. B., Huber, C. G., McAndrews, M. P. (2010). Hippocampal–neocortical networks differ during encoding and retrieval of relational memory: Functional and effective connectivity analyses. *Neuropsychologia*, *48*, 3272-3281.

Mechelli, A., Penny, W. D., Price, C. J., Gitelman, D. R., Friston, K. J. (2002). Effective connectivity and intersubject variability: using a multisubject network to test differences and commonalities. *NeuroImage*, *17*, 1459-1469.

Palmer, S. J, Eigenraam, L., Hoque, T., McCaig, R. G., Troiano, A., Mckeown, M. J. (2009). Levodopa-sensitive, dynamic changes in effective connectivity during simultaneous movements in Parkinson’s disease. *Neuroscience*, *158*, 693-704.

Peltier, S., Stilla, R., Mariola, E., LaConte, S., Hu, X., Sathian, K. (2007). Activity and effective connectivity of parietal and occipital cortical regions during haptic shape perception. *Neuropsychologia*, *45*, 476-483.

Périn, B., Godefroy, O., Fall, S., de Marco, G. (2010). Alertness in young healthy subjects: an fMRI study of brain region interactivity enhanced by a warning signal. *Brain and Cognition*, *72*, 271-281.

Rowe, J. B. (2010). Connectivity analysis is essential to understand neurological disorders. *Frontiers in Systems Neuroscience*, 4, doi: 10.3389/fnsys.2010.00144.

Schlösser, R., Gesierich, T., Kaufmann, B., Vucurevic, G., Hunsche, S., Gawehn, J., et al. (2003). Altered effective connectivity during working memory performance in schizophrenia: a study with fMRI and structural equation modeling. *NeuroImage*, *19*, 751-763.

Sehatpour, P., Dias,E. C., Butler, P. D., Revheim,N., Guilfoyle, D. N., Foxe, J. J., et al. (2010). Impaired visual object processing across an occipital-frontal-hippocampal brain network in schizophrenia. An integrated neuroimaging study. *Archives of General Psychiatry*, *67*(8), 772-782.

Sevel, L. S., Craggs, J. G., Price, D. D., Staud, R., & Robinson, M. E. (2015). Placebo analgesia enhances descending pain-related effective connectivity: a dynamic causal modeling study of endogenous pain modulation. *The Journal of Pain*, *16*(8), 760-768.

Shen, M.D., Shih, P., Öttl, B., Keehn, B., Leyden, K.M., Gaffrey, M.S., et al. (2012). Atypical lexicosemantic function of extrastriate cortex in autism spectrum disorder: evidence from functional and effective connectivity. *NeuroImage*, *62*, 1780-1791.

Shih, P., Shen, M., Öttl, B., Keehn, B., Gaffrey, M. S., Müller, R.A. (2010). Atypical network connectivity for imitation in autism spectrum disorder. *Neuropsychologia*, *48*, 2931-2939.

Sideridis, G., Simos, P., Papanicolaou, A., & Fletcher, J. (2014). Using structural equation modeling to assess functional connectivity in the brain: Power and sample size considerations. *Educational and Psychological Measurement*, *74*(5), 733-758.

Smith, S. M., Vidaurre, D., Beckmann, C. F., Glasser, M. F., Jenkinson, M., Miller, K. L., … (2013). Functional connectomics from resting-state fMRI. *Trends in Cognitive Sciences*, *17*(12), 666-682.

Steele, J. D., Meyer, M., Ebmeier, K. P. (2004). Neural predictive error signal correlates with depressive illness severity in a game paradigm. *NeuroImage*, *23*, 269-280.

Stein, J. L., Wiedholz, L. M., Bassett, D. S., Weinberger, D. R., Zink, C. F., Mattay, V. S., ... (2007). A validated network of effective amygdala connectivity. *NeuroImage*, *36*, 736-745.

Stricker, J. L., Brown, G. G., Wetherell, L. A., Drummond, S. P. A. (2006). The impact of sleep deprivation and task difficulty on networks of fMRI brain response. *Journal of the International Neuropsychological Society*, *12*, 591-597.

Stroman, P. W., Khan, H. S., Bosma, R. L., Cotoi, A. I., Leung, R., Cadotte, D. W., & Fehlings, M. G. (2016). Changes in pain processing in the spinal cord and brainstem after spinal cord injury characterized by functional magnetic resonance imaging. *Journal of Neurotrauma*, *33*(15), 1450-1460.

Szabo, C.A.; Salinas, F.S.; Li, K.; Franklin, C.; Leland, M.M.; Fox, P.T.; Laird, A.R.; & Narayana, S. (2016). Modeling the effective connectivity of the visual network in healthy and photosensitive, epileptic baboons. *Brain Structure & Function*, *221*(4), 2023 - 2033.

Szatkowska, I., Bogorodzki, P., Wolak, T., Marchewka, A., Szeszkowski, W. (2008). The effect of motivation on working memory: An fMRI and SEM study. *Neurobiology of Learning and Memory*, *90*, 475-478.

Taniwaki, T., Okayama, A., Yoshiura, T., Togao, O., Nakamura, Y., Yamasaki, T., et al. (2006). Functional network of the basal ganglia and cerebellar motor loops in vivo: Different activation patterns between self-initiated and externally triggered movements. *NeuroImage*, *31*, 745-753.

Taniwaki, T., Okayama, A., Yoshiura, T., Togao, O., Nakamura, Y., Yamasaki, T., et al. (2007). Age-related alterations of the functional interactions within the basal ganglia and cerebellar motor loops in vivo. *NeuroImage*, *36*, 1263-1276.

Toni, I., Rowe, J., Stephan, K.E., Passingham, R.E. (2002). Changes of Cortico-striatal Effective connectivity during Visuomotor Learning. *Cerebral Cortex*, *12*, 1040-1047.

Tourville, J. A., Reilly, K.J., Guenther, F.H. (2008). Neural mechanism underlying auditory feddback control of speech. *NeuroImage*, *39*, 1429-1443.

Unsworth, N., Fukuda, K., Awh, E., & Vogel, E. K. (2015). Working memory delay activity predicts individual differences in cognitive abilities. *Journal of Cognitive Neuroscience*, *27*(5), 853-865.

Vitali, P., Tettamanti, M., Abutalebi, J., Ansaldo, A.I., Perani, D., Cappa, S.F., ... (2010). Generalization of the effects of phonological training for anomia using structural equation modelling: A multiple single-case study. *Neurocase*, *16*(2), 93-105.

Whalley, L.J.; Staff, R.T.; Fox, H.C.; & Murray, A.D. (2016). Cerebral correlates of cognitive reserve. *Psychiatry Research Neuroimaging*, *247*, 65 - 70.

Zhuang, J., LaConte, S., Peltier, S., Zhang, K., Hu, X. (2005). Connectivity exploration with structural equation modeling: An fMRI study of bimanual motor coordination. *NeuroImage*, *25*, 462-470.

Zhuang, J., Peltier, S., He, S., LaConte, S., Hu, X. (2008). Mapping the connectivity with structural equation modeling in an fMRI study of shape-from-motion task. *NeuroImage*, *42*, 799-806.
